# A Rationale for a Gamified E-Coach Application to Decrease the Consumption of Sugar Sweetened Beverages

**DOI:** 10.3389/fdgth.2020.564529

**Published:** 2021-01-18

**Authors:** Robbert Jan Beun, Claire Luiten, Chris Verbeek, Maartje P. Poelman

**Affiliations:** ^1^Department of Information and Computing Sciences, Utrecht University, Utrecht, Netherlands; ^2^Department of Human Geography and Spatial Planning, Faculty of Geosciences, Utrecht University, Utrecht, Netherlands; ^3^Faculty of Science, Informatics Institute, University of Amsterdam, Amsterdam, Netherlands; ^4^Chair Group Consumption and Healthy Lifestyles, Wageningen University & Research, Wageningen, Netherlands

**Keywords:** behavior change, design patterns, gamification, e-coaching, sugar sweetened beverages, adolescents

## Abstract

The design and implementation of applications for behavior change should be preceded by careful analysis of the behavior change process and the target population. We, therefore, present on the basis of a blended research approach a rationale, opportunities and basic requirements for an application that offers a program for reducing intake of sugar sweetened beverages (SSB) by adolescents. This paper discusses the role of e-coaching and gamification as two high-touch design patterns in the behavior change process. Both design patterns aim at supporting the individual in a transformational journey from a current state toward a desired state where the detrimental behavior should be replaced by healthy alternative behavior. First, an elementary behavior scheme is introduced that frames three empirical studies. In the first study (plenary focus groups; *n* = 13), participants advised to include system recommendations for alternative healthy behavior, stressed the need for personalization of the e-coach and showed strong appreciation for the inclusion of gamification elements. The second study (online survey; *n* = 249) showed that SSB-intake is highly contextual and that reasons for (limiting) consumption SSB varies greatly between individuals, which the e-coach application should take into account. In a final small-scale pilot study (*n* = 27), we observed the potential of the inclusion of gamification elements, such as challenges and rewards, to increase compliance to the self-monitoring process of SSB consumption. Building upon these insights and prior studies, we argue that an e-coach mimics the collaborative practice of the program; its main task is to enrich the interaction with cooperative conversational experiences, in particular with respect to the alignment between user and system, motivational encouragement, personalized advice, and feedback about the activities. In addition, we outline that gamification not only has the potential to increase self-monitoring of the target behavior, user engagement, and commitment with the intervention program, but also enables a designer to shift long-term negative outcome of excessive intake in real life to short-term consequences in a virtual environment. In future larger follow-up studies, we advise to integrate the two design patterns within a social network of virtual and human agents that play a variety of competitive, normative and supportive roles.

## Introduction

Nowadays, the use of internet and smartphones has become near universal and provides the opportunity to deliver digital health interventions via new channels. Mobile internet technology reaches a large number of people, minimizes the need for face-to-face appointments, can be used anywhere and anytime and can overcome barriers to visit a coach in real life. To illustrate the latter, a majority of the Dutch youth (56%) finds online communication easier than face-to-face contact; in addition, a substantial number (38%) feels more comfortable to communicate using digital tools (1). Also, and in contrast with mass media and desktop applications, smartphone technology offers a dynamic and contextualized environment that enables powerful persuasive strategies, such as the assessment of relevant momentary information and the delivery of fully automated feedback.

Despite the numerous availability of technology based health-promotion systems, smartphone applications that are theory and evidence based are scarce (2–5). In addition, compliance to the various activities in behavior-changing programs is an ever-present problem (6, 7), in particular in those cases where human advisors and therapists are replaced by an automated digital version. This identifies the need to develop persuasive applications via a design process that carefully analyzes and evaluates the context of health behavior in combination with the needs, wishes and abilities of the target group.

In this paper, we focus on the process of reducing the intake of sugar sweetened beverages (SSB) by adolescents. Reducing SSB consumption is important, because it is related to an excess of weight gain in children (8, 9). Moreover, it is associated with diet-related obesity and diet-related chronic diseases later in life. On average, 30% of children's sugar intake can be assigned to the consumption of SSB (10). The consumption of SSB increases by age into adolescence (11). Also, social disparities are visible: children from families with a lower socio economic position (SEP) consume more frequently SSB compared to children from families with a higher SEP (10–13).

Worldwide public health efforts are in place to support individuals to reduce SSB consumption, varying from environmental (e.g., soda tax, labeling) to individual-focused interventions (e.g., educational intervention programs or campaigns). Key in individual-focused interventions is the integration of behavioral change techniques to improve the individuals' ability to change their target behavior (e.g., lower SSB consumption), but also to keep users motivated to remain involved with the program (14, 15). An effective form of an individual-focused intervention to improve health behaviors is “personal coaching” (16). Central techniques in personal coaching are, for instance, shared goal setting, action planning, self-monitoring of the target behavior and receiving feedback (17).

The aim of this paper is to present a rationale, opportunities and basic requirements for a gamified e-coach application that offers a program to support adolescents in their behavior change process. Prior studies emphasize that the use of gaming elements combined with the power of natural conversations of a supportive e-coach aligns with the interaction paradigms and preferences of the envisaged target population: among this group, gaming, and text messaging are popular activities performed by a smartphone (18, 19). E-coaching systems exist in many forms and functions, and may be targeted at a variety of behavior change domains (20–22). Current technology enables a persuasive system to mimic the conversational process of an active partner that not only creates awareness about the target domain and the process of change, but that also empowers the system to offer a coherent and tailored program for change and to build a social relationship with its user (20, 23). Gamification, which is defined as the use of game design elements in a nongame context (24), has the potential to increase engagement and compliance with the program by elements such as rewards, challenges and leader boards (25–28).

Before the actual design takes place, however, we first need a thorough analysis of the behavior change process and active involvement of the target population in various stages of the design process (29). We target at the role of e-coaching and gamification as two high-touch design patterns that support the behavior change process and we try to substantiate the idea that both patterns enable the support of the individual in a transformational journey from a current state toward a desired state where the detrimental behavior has been replaced by healthy alternative behavior.

To get an early grip on the requirements and the functional role of the two design patterns, we present three empirical studies and prior international research on the basis of an elementary behavior change scheme. First, we discuss the scheme in Section Behavior change determinants and techniques and summarize the relevant background of the process of change in terms of the behavioral determinants. It is argued that a great deal of SSB consumption is performed on the basis of a rather unconscious process and that changing the behavior requires powerful and adequate persuasive techniques. In Section Empirical Studies, we report the results of the three empirical studies. First, to get a general understanding of user preferences and needs in the early design stage of the envisaged system, we present a qualitative study including (plenary) focus group sessions. During these sessions, participants' discussed motives for SSB consumption, various design elements and potential functionality. In the second study, we elaborate in a cross-sectional online questionnaire on the circumstances of the SSB consumption and the role of personalization. In particular, we investigate the circumstances, in terms of place, time and company, that increase or decrease the chance for SSB consumption, and the target group's motivation to consume SSB and their susceptibility for change. In the third study, we focus in a small-scale pilot experiment on one of the functional elements of the application: the self-monitoring of SSB consumption. Here, we were interested to see whether gamification enhances the self-monitoring process of SSB consumption via a smartphone. In Section The Role of E-Coaching and Gamification the functional roles of the e-coach and gamification in the behavior change process are discussed. In Section Discussion and Conclusions we conclude this paper with the main results and recommendations for future research.

## Behavior Change Determinants and Techniques

Attitudes, social norms and self-efficacy are behavioral determinants frequently related to SSB consumption and explain the *intention* to consume SSB by a variance of 34 to 61% (30, 31). The stronger people's intention, the more likely it is that the behavior will be engaged in (32). Yet, intentions can only partly explain behavioral decisions and do not always translate into the actual behavior; this is also called the “intention-behavior gap” (33). When repeated and learned activities become automatic responses to specific cues, such as location and time, they become habits. Because habitual action is detached from conscious control, the decision to act is intuitive and spontaneously triggered. Many dietary behaviors are habitual and, therefore, hardly without intention forming. For instance, in case of SSB consumption adolescents may automatically choose a beverage when watching television at night (34).

In general, habit forming is a complex process, including concepts such as learning and reward, but it has been shown that even the simple repetition of behavior may lead to an increase of the behavior (35). Old ingrained habits are difficult to break, however. Also, since habit formation involves some sort of reward system, the brain craves for the reward–taking away the habit, implies taking away the reward. Consequently, and in line with dual-process theories of dietary behavior [see e.g., (36)], we assume that a great deal of SSB consumption is performed on the basis of a rather unconscious processes. Therefore, changing health-compromising behavior requires first of all careful analysis of the process of change and powerful persuasive techniques to construct the intention for healthy behavior and to maintain the behavior in the future. We now first introduce a conceptual schema that frames the requirements for a system that offers support in reducing SSB consumption.

### The Cue-Behavior-Outcome Schema

A behavior change support system–or so-called persuasive system–may opt for a number of persuasive methods, varying from reminders and goal setting to various types of social pressure [e.g., (14, 37–39)]. Adequately applying these techniques, however, requires some understanding of the behavior change process. We will, therefore, first consider the basic elements of the schema of a habitual activity:^1^


$$\begin{array}{l} \text{cue}-\text{behavior}-\text{outcome} \end{array}$$

In theory, we can intervene in all three elements [c.f., Wendel (40)], i.e., avoid the cue, replace the old behavior, change the percept of the outcome, or a combination of these techniques.

Intentionally avoiding the cue requires first of all knowing what the cue is, i.e., what internal or external circumstances increase the chance to perform the behavior (e.g., time, event, emotion, physical condition). Secondly, the individual (or some other responsible) has to find methods to avoid the cue, for instance, removing the SSB vending machine from school (external) or reduction of stress (internal). In practice, however, many cues, such as dinner with family or hanging out with friends, are unavoidable or avoiding them may have a large impact on the individual's well-being. At the supporting system side a problem may be that the detrimental behavior–here SSB consumption–is outside its reach. So, often the system does not know when or where the person is engaged in the behavior and, therefore, lacks a sense of urgency to adequately respond to the relevant dynamics of the situation.

Replacing the behavior implies that the original cue ensures that the chance to perform the alternative behavior is greater than the chance to perform the old behavior. Now, the impulse to perform the original behavior should be suppressed and an alternative behavior should be put in place. This is especially difficult for habits, and requires a switch from the intuitive system to conscious planning and frequent repetition of the alternative activity. An important aspect is that both motivation and ability to perform the alternative behavior must be sufficiently high–in other words, the returns of the alternative behavior should be in proportion to the investments. An example could be the replacement of SSB with sparkling water (without sugar) to quench thirst (the final goal or reward).

The outcome often represents some type of reward, for instance, quenching thirst or joy of taste of SSB consumption, and as a result the craving for SSB stops and results in relaxation on the short-term. But clearly, health-compromising habits always have detrimental side-effects, in particular in the long run (e.g., chronic diseases, caries), and the individual may be unaware of these risks later in life. So, a strategy the persuasive system may apply is making the person aware of the unavoidable long-term and health consequences. In practice, however, this may not be sufficient for various reasons, e.g., the person believes he/she will get away with it (e.g., optimistic bias) or prefers the short-term experience (taste) over the long-term consequences. An additional strategy, therefore, would be to shorten the delay of a negative outcome, for instance, by real-time negative assessment of friends or a virtual character.

In line with, for instance, the transtheoretical model (TM) by Prochaska and Velicer (41), we consider behavior change as a process that requires various stages to rationalize the old and the new behavior, and to put the new behavior in place of the old one. The process consists of at least the following stages and substages (below we will use α for the old behavior and α_alt_ for the new behavior):

1. Raising awareness of α and its negative consequences (pre-contemplation stage in TM)
2. Changing the behavior from α to α_alt_:
  - Accepting to abandon α (contemplation stage in TM)
  - Establishing commitment to do α_alt_ (preparation stage in TM)
  - Raising awareness of the cues (contemplation/preparation stage in TM)
  - Practicing α_alt_ (preparation stage in TM)
  - Repeating α_alt_ (action stage in TM)
3. Maintaining α_alt_ (maintenance stage in TM)

Although the resulting states of these stages are necessary pre- and post-conditions in the change process, different activities of the (sub)stages may run in parallel. For instance, full acceptation to abandon α (2a) and commitment to perform α_alt_ (2b) may require the individual's experience of the investments for the alternative (2d). Moreover, the target behavior usually consists of many subactivities (e.g., buying SSB, putting it into or taking it out of the fridge, opening the bottle, take a glass, put it on the table, pour it into the glass, …) and, as a result, the stages may differ for various subactivities.

To apply adequate persuasive strategies, the supporting system should align as much as possible with the user and become familiar with a blend of user characteristics, such as stage of change, motivation to drink, daily consumption, company and time of the day, needs, and preferences. System awareness of the cues for the behavior may be important, not only because it enables the application to raise user's awareness about the cues in stage 1 of the behavior change process, but also to intervene at the user's weakest moments. Also, to target the correct user intentions and beliefs, system awareness of personal motivations for intake or reduction may be important. Consequently, optimal support requires the construction of some sort of common ground and profound knowledge of the system about the user.

In all stages of the change process, a key component of such a system is a reliable monitoring tool to register the behavior of interest. In the ideal case, the process of monitoring could, and probably should, be automated, but given the current state of technology, users of these systems have to do at least some the work themselves. We know from previous research that compliance of adolescents with self-monitoring appears to be low (42), but current technology may have great potential for monitoring food intake activity among adolescents (43), in particular when the activity becomes an enjoyable and challenging experience. This is an example of one of the many places where gamification elements may be included that offer non-delayed rewards right after the performance of the activity.

These considerations raise questions such as what type of information should be presented in the common ground, how can the information be applied in a gamified e-coaching system and what type of gamification elements can be used to optimize the change process? Current literature provides some, but only limited insight. For example, energy drink consumption increases among college students especially when partying (44), when purchasing foods during school time (45) and being at specific places out-of-home (46). Block et al. studied reasons for students to consume SSB; price and taste were mentioned frequently (47). To our knowledge, however, understanding of individuals' rationale for less SSB consumption is unknown. To uncover at least some of the relevant knowledge, we will now turn to the empirical studies.

## Empirical Studies

### Study 1: User Group Preferences and Needs

#### Study Design, Procedure, and Measures

To get a general understanding of user preferences and needs, a qualitative study was conducted including a plenary focus group session and a World Café method. The World Café method is mimicking a café consisting of small tables, each representing a sub-issue or sub-question for discussion. Smaller groups rotate every 20 minutes so that each sub-group is able to participate in each issue or question. The advantage of the World Café method is that it enables participants to share and generate new ideas upon the basis of the previously generated ideas of earlier participants (48).

To recruit participants, ten vocational education schools were approached with the question to participate. Schools received a flyer by e-mail with information on the project. Of the eight schools that replied, only one school (Wellant college in Rijswijk, the Netherlands) was willing and able to participate with one class.

##### Plenary Focus Group Session

By means of a short plenary focus group session (~30 min.), we introduced the session, engaged the participants with the topic and tried to gain insight in their motives for SSB consumption. In doing so, we incorporated three “personas” which were representations of fictitious target users. Using personas in user-centered design processes has many benefits (49): increase of the participants' identification with situations in which SSB consumption is common, but also stimulation of the participants to come up with ideas or solutions for the fictitious personas. These personas were introduced on a digital screen and the interviewer asked questions, such as “Why would Persona 1 drink an energy drink after his work-out?” and “What would you advise Persona 2 to drink and why?” In addition, we identified the SSB consumption of the participants (e.g., “What do you usually drink at school?”) and about their ideas of drinking non-sugary drinks (e.g., “How could drinking non-sugary drinks be more attractive?”).

##### World Café Method

After the plenary session, we organized small-group discussions according to the World Café method (48) creating three sub-groups. At each table in the World Café session, the researchers (CL, CV) and an involved research assistant chaired and guided the discussions, including one of the following three topics: “the e-coach,” “gamification,” and “general design.”

**Figure 1:**
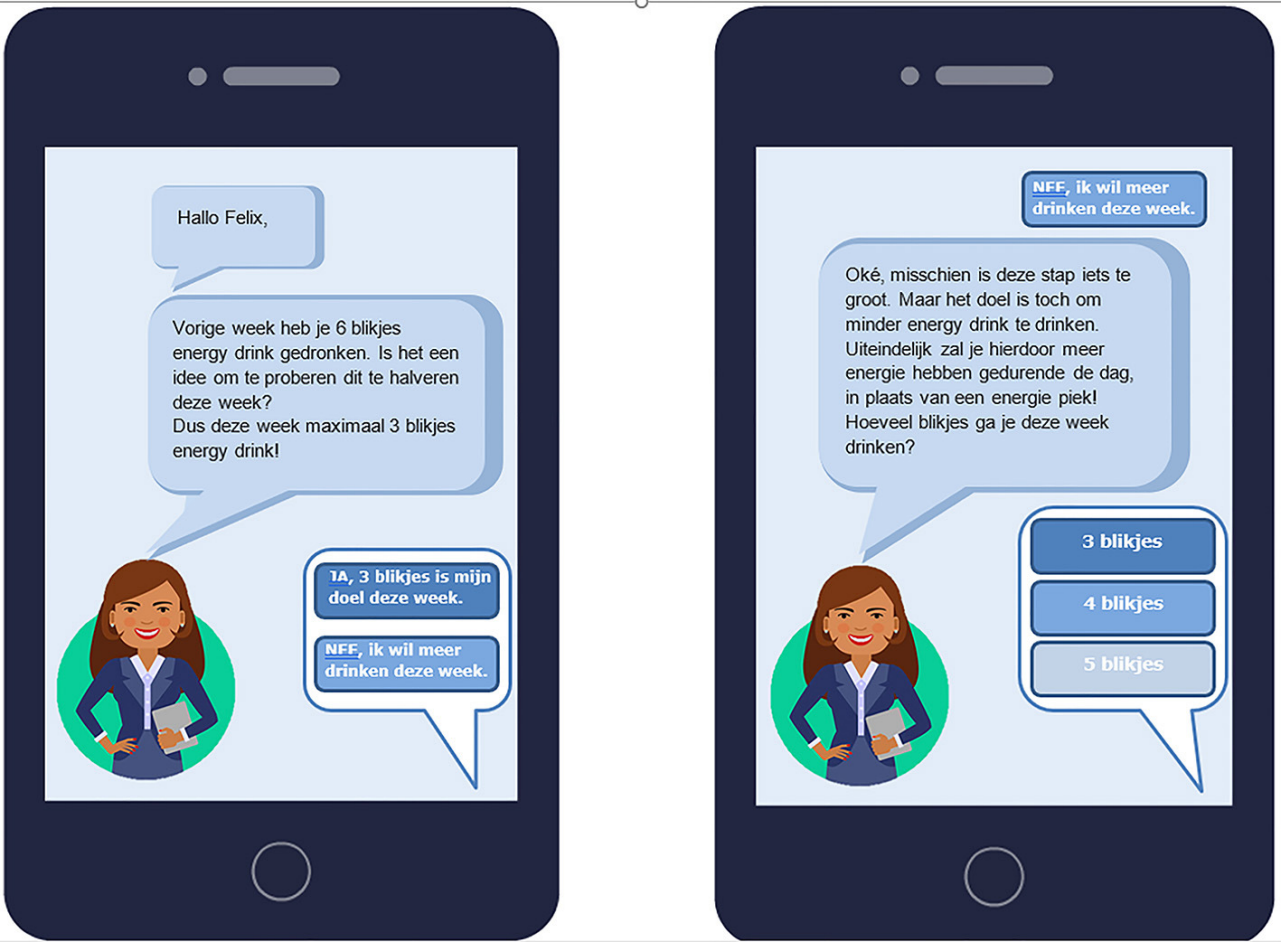
Example of the e-coach interface used during the World Café sessions.

Table A–The e-coach: At this table, the participants' imagination of an e-coach was stimulated. We presented participants with printed screen shots of a fictional participant with a fictional e-coach (see Figure 1). The following research question was discussed: “What are the needs and requirements regarding the e-coach element in the application?” Each participant was asked to write down positive and negative characteristics of the e-coach. Subsequently, the participants were stimulated to discuss their thoughts and to provide suggestions that an e-coach should include to be attractive to use.

**Figure 2:**
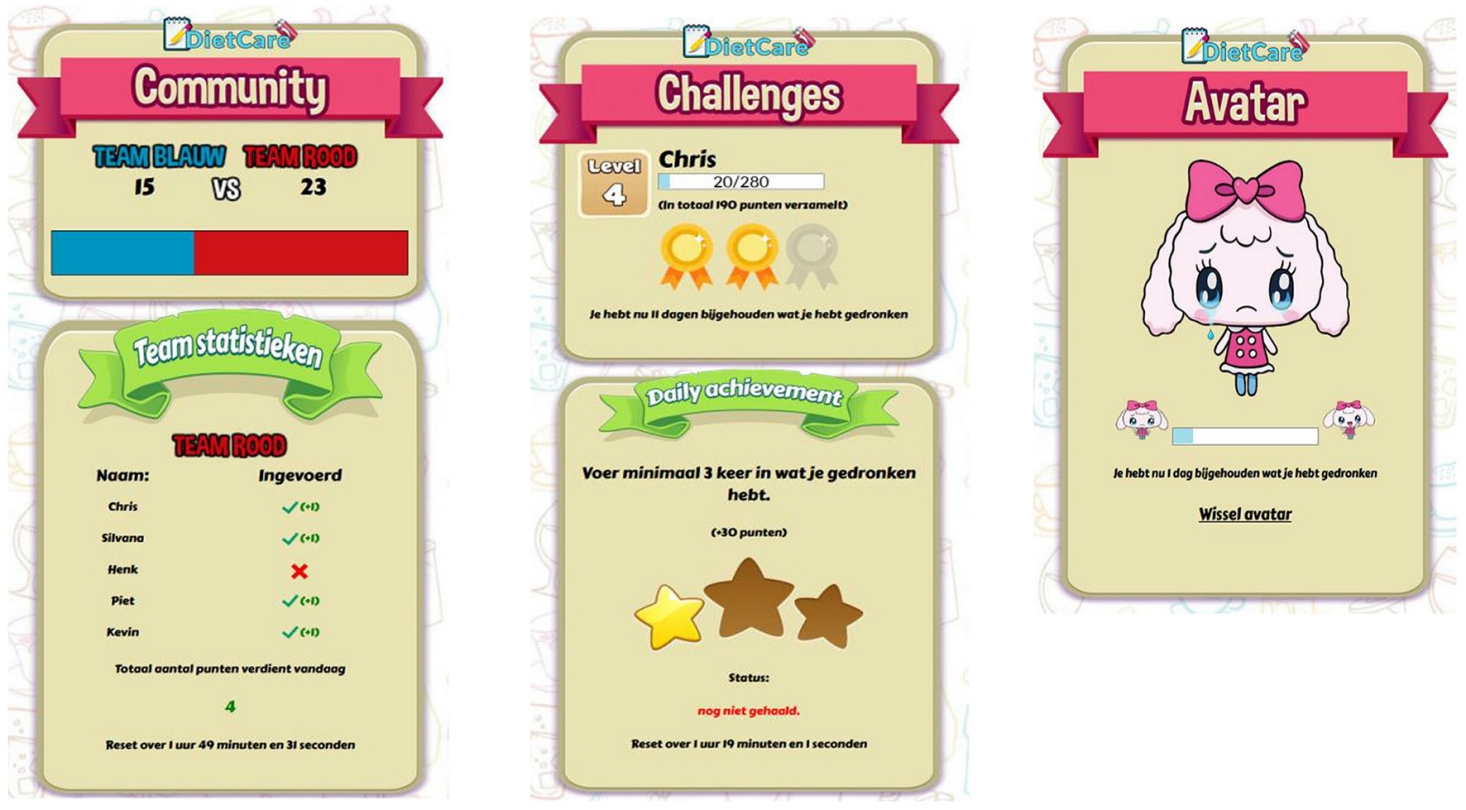
Example of the social prototype (left), challenges prototypes (middle), and avatar prototype (right). The social prototype keeps track of team statistics, in this case Team Red, and users can observe the self-monitoring activities of other users. The challenges prototype shows a reward in terms of points, stars, and medals based on the entered drinks. The avatar prototype shows positive or negative emotions on the basis of the entered drinks.

Table B–Gamification: At this table, participants discussed different gamification elements. The following research question was discussed: “Which gamification elements are appealing to you and why?” Three different prototypes of a self-monitoring application for SSB-intake were presented. Participants could play with the prototypes on a smartphone or could comment on it by using printed materials (e.g., screenshots of the gamified self-monitoring prototypes). Each prototype contained a different element of gamification (see Figure 2): (1) a challenge-and-reward prototype by which users received points when using the self-monitoring application, (2) an avatar prototype that resulted in a happier avatar when beverages were monitored (and “sad” vice versa), (3) a social prototype where participants could observe and compare how other users monitored their beverage consumption.

Table C–General design: At this table, participants were stimulated to use their creativity to design their ideal application. The following research question was discussed: “What are the needs and requirements regarding the design of the application?” Participants could draw and write down their suggestions on an A1 flipchart paper. The chair asked questions to clarify the participants' design requirements and steer their input in the requested direction.

##### Analyses

The discussions during the focus group and the World Café sessions were recorded and transcribed afterwards.

#### Results

##### Participants

In total, 13 students of one class participated (*n* = 7 female, *n* = 6 male). Their age ranged between 16 and 27. To keep the participants' required effort to a minimum, the meeting took place at their school.

##### Focus Group Session

The following five determinants were mentioned in relation to beverage consumption: *price, availability, habit, taste*, and *convenience*. Reasons to choose for SSB were often related to “waking up in the morning” or “boosting one's energy.”

“*I had an energy drink this morning, because I did not sleep well last night.” (male, age 18)*.

Some participants perceived SSB consumption as a habit, where a period of “rehab” would be required. Furthermore, specific social norms for drinking SSB were identified during the conversation, such as “Everybody drinks coffee in the morning to wake up” and “Drinking water with fruit is not cool.” Many participants were, however, aware of the sugar content in different drinks:

“*Some fruit drinks might contain even more sugar than soda drinks.” (female, age 20)*.

Drinking water was also mentioned and the participants indicated to bring water from home because it is easy, for free and healthy. In addition, participants mentioned that water is “nice” to drink if you are thirsty.

In the discussion with participants, two approaches were proposed by the participants to make drinking non-sugar sweetened beverages more popular. The first was a *reward-system* as possible strategy, for example, points could be earned and returned for rewards. The second approach was promoting the application with help of *social influencers*. Participants agreed that “YouTubers” could play an important role as they follow them on a daily basis. Furthermore, some participants considered adding fruit into drinking water as a viable strategy to make water tastier. This contradicted the viewpoint, however, that drinking fruit-water is not “very cool.”

##### World Café

Table A–The e-coach: The majority of participants liked the idea of having an avatar as e-coach. Although we only presented participants with a fictional e-coach, some participants experienced the e-coach as too strict, whereas others opted the function to choose their own avatar. Participants like to choose how the avatar looks like [e.g., the appearance (sportive, professional), outfit, or sex] and the tone of text messaging [e.g., direct, (in)formal]. Furthermore, participants agreed on having a conversation with the e-coach once a day, because more conversations would make it “*annoying*.” One individual stated, “*The start of a conversation with the e-coach could be notified with the help of a red circle, comparable to the ones used in other applications*.” (male, age 18).

Table B–Gamification: The three gamified self-monitoring prototypes were positively perceived and the participants believed they would stimulate them to submit their consumptions during the day. Most participants preferred the prototype with the challenge-and-reward system over the avatar and the social prototype. They indicated that this would encourage them to track their consumption, especially when the points would give the opportunity to get to a *next level*. Participants also proposed to include the option to choose their game-element of interest. Moreover, participants suggested to send notifications when beverage monitoring is required, rather than asking to monitor SSB-intake at set times.

Table C–General design: Several participants proposed the option of adding a *database* to the application with healthy alternatives for SSBs or recipes for healthy drinks. Talking about this topic, one participant commented: “*I think a glass of water with a leaf of mint is experienced as more attractive*.” (male, age 23). While designing their ideal application, the participants mentioned the possibility to visualize the amount of sugars in drinks and to show the submitted data on consumed drinks in a comprehensibly manner. Two of the three groups at this table proposed the possibility to adapt the application to their personal preferences.

#### Conclusion

These focus group sessions should be considered as a first attempt to obtain qualitative information about user needs and preferences in an early design stage. Participants of the sessions endorsed the inclusion of gamification elements and a supporting avatar in the role of an e-coach. In the World Café sessions a preference was expressed for the “challenge-and-award' prototype”; participants emphasized, however, the inclusion of an option to choose the gamification elements by themselves. In general, participants stressed the need to control settings of the application, such as the number of reminders, and the appearance and tone of voice of the avatar. Another prominent demand is feedback about the user's past performance of both the old and alternative behavior in terms of the amount of sugar or submitted drinks. Finally, participants suggested the inclusion of a database with recipes and recommendations for healthy alternative behavior (e.g., water with mint or fruit).

From this impressionistic overview, there are many ways to continue and many research questions to be answered before the actual design should start. In the next two empirical studies, we opted for the following questions: first, which cues or situational factors increase the chance of SSB-intake and what are motivations for consuming and reducing SSB-intake? (Study 2) and second, does gamification support self-monitoring SSB-intake in real world settings? (Study 3).

### Study 2: Contextual Factors Aligned With Sugar Sweetened Beverage Consumption

#### Study Design, Procedure, and Measures

To uncover the circumstances of the SSB consumption and the target group's motivation to consume SSB and their susceptibility for change, a cross-sectional online questionnaire study was conducted among Dutch adolescents and young adults (<27 years). In the Netherlands, the educational system comprises three levels, based on the intellectual abilities of students: pre-vocational, senior general, and pre-university. All of the adolescents participating in the current study were following or completed the pre-vocational track. A third-party commercial contractor, PanelClix, recruited students via email and invited them to fill out the survey (survey items available upon request). After completing the questionnaire, the participants were rewarded with PanelClix-points.

First, demographic variables were assessed, including sex, age, and ethnic descent. Moreover, self-reported height and body weight were obtained and body mass index (BMI, kg/m^2^) was calculated. Second, consumption frequency [glasses (250 mL) per week] of SSB and water were determined using the validated Brief Questionnaire to Assess Habitual Beverage Intake (BEVQ-15) (50). Third, Multiple choice questions about contextual factors of SSB consumption, including multiple response options for place (e.g., at school, home), company (e.g., alone, classmates), and time (e.g., morning, afternoon) and mealtime (e.g., at breakfast, lunch). Participants could click as many answers as possible. Finally, set motivations to consume SSB were questioned by means of seven statements, which could be answered on a 7-point Likert scale, ranging from 1 (really unimportant) to 7 (really important). For example, ‘*It is important to me that the SSB I consume is tasteful*’. Fourth, individual reasons to decrease SSB consumption were questioned, using a 5-point Likert scale ranging from 1 (disagree) to 5 (agree). The statements were deduced from existing questionnaires on motives for alcohol consumption amongst adolescents (51, 52).

##### Analyses

Demographic characteristics, self-reported SSB consumption, motivations to drink SSB and reasons to quit SSB were obtained using descriptive statistics. Mean beverage per week was examined by multiplying the frequency of consumption (times per week) by the amount of glasses consumed. Subsequently, the following three SSB-consumption groups were calculated (1) ≤ 1 glass/per week (low consumption); (2) 1 < 7 glasses/week (medium consumption); (3) ≥ 7 glasses/week (high consumption). The proportions of multiple response variables were compared between low, moderate and high consumers by estimating the variance for the categories compared, including Bonferroni-corrections to account for multiple comparisons using IBM SPSS V25.0.

#### Results

##### Participants

In total, 249 participants with a lower vocational education completed the survey and were included in the analyses. Their mean age was 21.4 years (*SD* = 3.4), 49.8% was female and the majority (95%) had a Dutch ethnic background. In total, 239 participants (96%) filled out the questions on height and body weight. Their mean self-registered BMI was 23.95 kg/m^2^ (*SD* = 5.1).

##### Beverage Intake

Among the total sample, water was the most consumed daily (median = 750 mL, IQR = 750). The median of daily soda intake (containing sugar) was 250 mL (IQR = 375). Energy drinks (median = 125, IQR = 250) and non-sugary sodas (median = 125 mL, IQR = 250) were least consumed. In total, 34.5% of the participants indicated to consume seven of more glasses of SSB per week whereas 35% consumed less than one glass (250 mL) of SSB per week.

##### Contextual Factors of SSB Consumption

Table 1 presents the contextual factors by low, medium, or high SSB consumption; in this paragraph only statistically significant differences between groups (*p* < 0.05) are discussed. High consumers consumed more frequently sugar sweetened beverages in the morning and afternoon compared to low and moderate consumers. Moderate consumers also consumed more often SSB in the afternoon than low-consumers. The more SSB consumed, the higher the likelihood to consume SSB during mealtime; especially during lunch and dinner, high consumers were significantly more likely to consume SSB compared to low and moderate consumers. High consumers consumed more often SSB at home compared to low and moderate SSB consumers. High consumers also more often consume SSB while commuting and at school compared to low consumers. Both moderate and high consumers consumed more SSB at work compared to low consumers. High consumers more frequently consume SSB when alone than low and moderate consumers.

**Table 1:** SSB consumption (%yes) per category for different situations [highest scores for each subgroup (low, moderate, or high-consumer) in italics].

**Contexts**		**≤1 glass per week (*n* = 87) (low-consumers)**	**>1, <7 per week (*n* = 76) (moderate-consumers)**	**≥7 glasses per week (*n* = 86) (high-consumers)**
Part of the day	Morning	4.5^a^	9.2^a^	24.4^b^
	Afternoon	28.4^a^	51.3^b^	71.1^c^
	Evening	*86.6^a^*	*78.9^a^*	*89.5^a^*
	Night	22.4^a^	15.8^a^	24.4^a^
Meal-time	At breakfast	0.0^x^	7.9^a^	11.6^a^
	At lunch	7.5^a^	18.4^a^	39.5^b^
	At dinner	31.3^a^	42.1^a^	*66.3^b^*
	Not	*67.2^a^*	*46.1^b^*	23.3^c^
Place	School	11.9^a^	22.4^ab^	30.2^b^
	Home	61.2^a^	*69.7^a^*	*88.4^b^*
	Sport club	6.0^a^	11.8^a^	10.5^a^
	Commuting	11.9^a^	26.3^ab^	38.4^b^
	At friends	*65.7^a^*	50.0^a^	65.1^a^
	Work	10.4^a^	31.6^b^	39.5^b^
	Club	40.3^a^	40.8^a^	34.9^a^
	Other	4.5^a^	2.6^a^	4.7^a^
Company	Friends	*83.6^a^*	*61.8^b^*	*80.2^a^*
	Parents	26.9^a^	30.3^a^	44.2^a^
	Classmates	16.4^a^	25.0^a^	30.2^a^
	Alone	31.3^a^	43.4^a^	72.1^b^
	Brother/sister	19.4^a^	26.3^a^	31.4^a^
	Colleagues	16.4^a^	18.4^a^	29.1^a^
	Other	6.0^a^	7.9^a^	8.1^a^

a, b, c *The same superscript letters indicate similar frequencies of SSB consumption during defined situations among the low, moderate and high consumers in the row (p > 0.05). For each pair of consumers (low, moderate, high), the proportions of multiple response variables were compared by estimating the variance for the categories compared, including Bonferroni–corrections to account for multiple comparisons*.

X *not used in the analyses as its column proportion is zero*.

##### Motivations to Consume SSB

Table 2 presents the motivations for SSB consumption, where “taste” is indicated as the most important motivation to drink SSB, followed by “quenching thirst.” The motivation to consume a drink “of a popular brand” was the lowest ranked motivation.

**Table 2:** Motives to drink SSB on a 7-point Likert scale (7 = very important).

**Motives**	**Mean**	**SD**
**“It is important for me that SSB is.”**		
Tasty	5.92	1.14
Quenching thirst	5.68	1.19
Price	5.06	1.38
Good feeling	5.03	1.44
Health	4.71	1.62
Balance weight	4.43	1.67
Popular brand	3.07	1.53

##### Reasons to Quit Drinking SSB

Table 3 presents reasons to quit SSB consumption. Becoming healthier in the long term (future), getting more energy of quitting, or if SSB would contain too much sugar were the most important reasons for participants to quit. To save money, behavior of others and a negative framing of SSB in the media were observed as less important reasons.

**Table 3:** Reasons to limit SSB consumption on a 5-point Likert scale (5 = very important).

**I would consume less SSB if**.	**Mean**	**SD**
I would become healthier in the long term	3.80	0.87
I would get more energy	3.73	0.94
It contains too much sugar	3.73	0.94
Is better for my teeth	3.69	0.91
I would feel healthier immediately	3.69	0.94
It would be unhealthy	3.68	0.97
It would make sick in the long term	3.66	1.02
It would make me look better	3.64	0.99
It would save me money	3.31	0.99
Nobody would drink it anymore	2.78	1.15
The media would negatively depict it	2.77	1.06

#### Conclusions

Compared to low- and moderate consumers, high-consumers most frequently indicated to consume SSB during lunch and dinner time, in the morning and afternoon, at school, at home, and while commuting. Also, they more frequently drank SSB while being alone. In line with study 1 and prior studies (47, 53), taste was the most important motive for SSB consumption. Reasons to quit SSB consumption were predominantly related to health. The majority of all participants consumes SSB at home and together with friends. So, in order to align a persuasive system to these contextual and user characteristics, the system requires special functions and tools to measure the environment (e.g., location data, company) or question the user by means of (simple) conversational structures. We will return to this in Section The Role of E-Coaching and Gamification.

### Study 3: Gamification for Self-Monitoring SSB Consumption

#### Study Design, Procedure, and Measures

**Figure 3:**
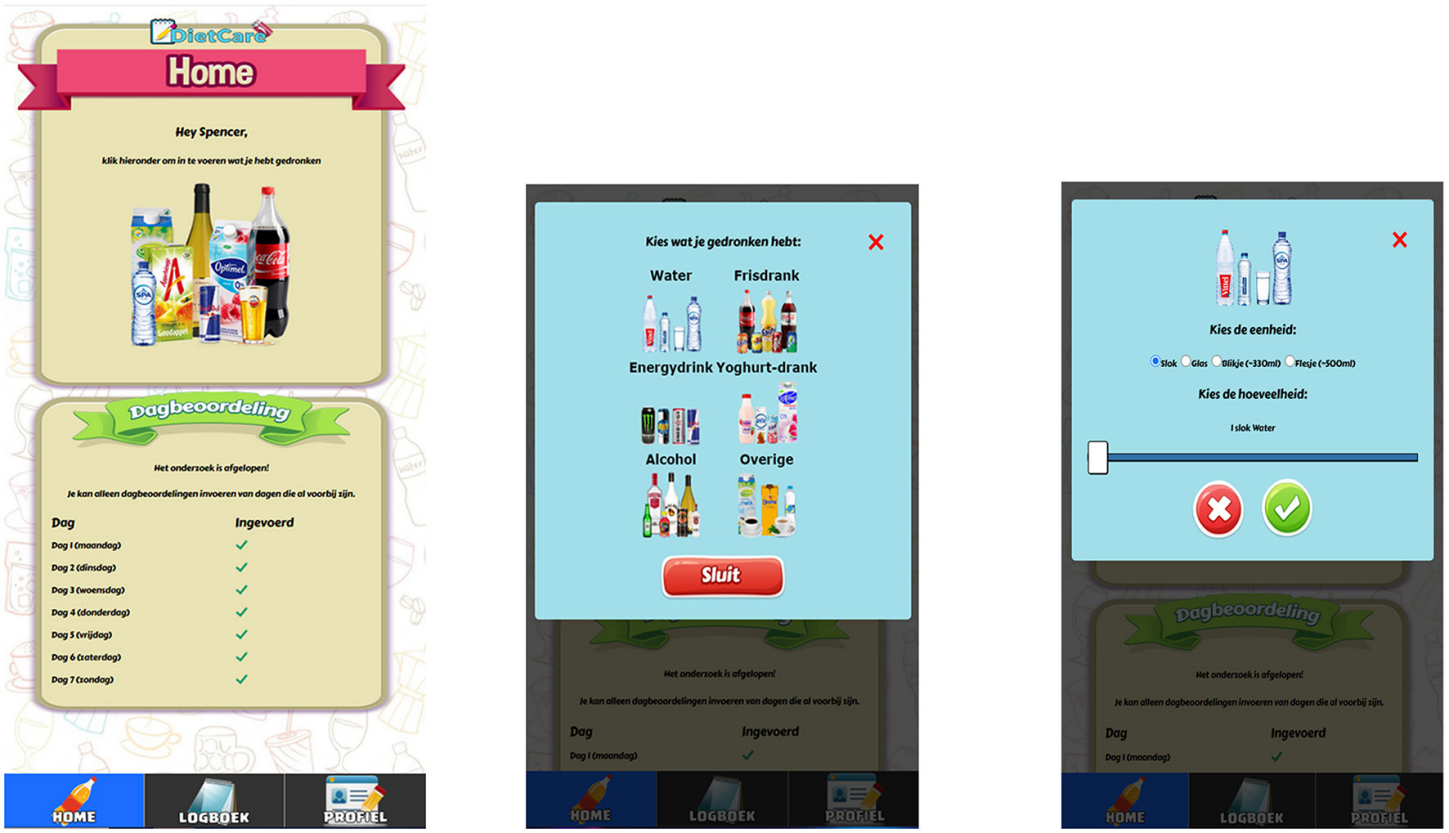
Three screenshots of the control prototype. The left picture shows the home page where drinks can be entered (top) and an overview of previous self-monitoring activities (bottom). The other two pictures show pops up after touching the top of the home page. It shows a selection of beverage types from which the user may choose (middle picture) and the amount of the selected type (right picture). Notice that this prototype does not contain gamification elements as in Figure 2.

To explore whether gamification enhances the self-monitoring process of SSB consumption, a small-scale randomized-control pilot study was conducted assessing the potential of three gamified self-monitoring prototypes against a control prototype. Here we used the three prototypes from Table B in Study 1 (social, challenge-and-reward and avatar; see Figure 2) and a control prototype (Figure 3).

A potential pitfall of stimulating the self-monitoring of SSB consumption is the possibility of stimulating the consumption. Therefore, the prototypes focused on monitoring *all* types of drinks, e.g., water, tea, diet drinks, irrespectively of the sugar content.

Adolescents and young adults (between 17 and 26 years old) were recruited by visiting four vocational education schools in the Netherlands. First, one of the researchers (CV) approached a random sample of students to ask them to participate. Next, a snowball sampling approach was used to invite more participants via the already assigned participants.

Participants received a link to download the self-monitoring application including one of the four prototypes (random assignment) and were requested to use the application for a one-week period. After one week, participants received a questionnaire to evaluate their attitude and opinion about the prototype.

The number of beverage submissions was automatically monitored by the smartphone application over a one-week period. After completing the monitoring period, participants received the online survey about their attitudes and opinion about the prototype. First, three items that could be answered on a 5-point Likert scale were used to determine if the application encouraged participants to monitor their SSB consumption accurately: “The application encouraged me to….” a. “add my drinks in the application,” b. “add my drinks frequently,” and c. “add my drinks accurately,” ranging from “totally not” (1) to “absolutely yes” (5). Moreover, participants were asked to provide a grade on a scale ranging from 1 to 10, for the prototypes' capability to stimulate beverage intake and overall liking.

##### Analyses

A mean (SD) score for encouragement was calculated based on the three items. Because of the small-scale nature of this pilot study, we only examined descriptive statistics for the overall number of beverage submissions, encouragement and the grading of the prototypes capability to stimulate beverage intake.

#### Results

##### Participants

Initially, 35 participants signed up for the study; 27 participants (77.1%) completed the online survey and were included in the analysis. Participants were on average 20 (SD 2.1) years old and 70% of the participants was female (Table 4).

**Table 4:** Demographic characteristics of the participants per condition.

**Condition**		**Sex**		**Age**	
	**Total (*N*)**	**Female %**	**Male %**	**Mean**	**SD**
Control	7	86	14	20.9	2.1
Challenge & rewards	7	71	29	21.4	1.4
Avatar	5	80	20	20.8	3.9
Social	8	50	50	19.5	1.8
Total	27	70	30	20.6	2.3

##### Beverage Submissions and Scores

The mean number of beverage submissions was 18.7 (SD 9.8) for the control application, 20.6 (*SD* = 10.0) for the social prototype, 30.1 (*SD* = 8.5) for the challenge-and-rewards prototype, and 26.0 (*SD* = 9.9) for the avatar prototype (Table 5). On the 1–5 scale, control group participants rated the encouragement of the prototype slightly lower than the other prototypes (3.0, *SD* = 0.7), where the difference was largest with the avatar prototype (3.6, *SD* = 0.8). Participants that used the challenge and rewards prototype gave the highest score (7.3) for its capability to stimulate monitoring of beverage submissions compared to the control group (5.9). The overall rating of the gamified prototype applications range between 7.0 and 8.0, where the control application was rated lower with a mean score of 6.4.

**Table 5:** Beverage submissions, mean rating on feeling stimulated to submit and the attitude toward the application stimulation.

**Condition**	**Beverage submissions**		**Encouragement proto-type** **stimulation (1–5)**		**Attitude stimulated** **by application (1–10)**		**Overall rating (1–10)**	
	**Mean**	**SD**	**Mean**	**SD**	**Mean**	**SD**	**Mean**	**SD**
Control	18.7	9.8	3.0	0.7	5.9	1.3	6.4	0.8
Challenge & rewards	30.1	8.5	3.2	0.9	7.3	1	7.3	1.0
Avatar	26.0	9.9	3.6	0.8	7	0.8	8	0.8
Social	20.6	10.0	3.3	0.8	7	0.9	7	0.8

#### Conclusion

Based on descriptive statistics, the results suggest that, compared to the control prototype, the gamified prototypes provoked more beverage submissions and that users felt more stimulated to do so. However, these outcomes should be interpreted with caution. Due to the small sample size and the absence of statistical testing, we cannot simply conclude that gamification helps. In our case, the game type was randomly assigned to the subjects, but the assignment probably requires some type of personalization based on, for instance, Marczewski's model of user types (54). This would be in line with the outcome of Study 1, where the subjects in the Table B session gave their preference to the challenge-and-award prototype and to personalization of the game type. So, these results give at least some direction for future research and the design of the application. In a follow-up study, a larger sample should be included to test statistical significance and to study the long-term utility of gamification elements to self-monitor beverage intake.

## The Role of E-Coaching and Gamification

We will now discuss how e-coaching and gamification elements can possibly support the reduction of SSB consumption and how the results of the previous studies may contribute to the design of such systems. Remember from Section Behavior change determinants and techniques that behavior change requires powerful persuasive techniques to construct the intention for healthy behavior and to maintain the behavior in the future, in particular in the case of detrimental habits. We, therefore, considered the application as a persuasive system that supports the behavior change process through different stages of change.

To increase the power of a persuasive system there may be important complementary roles for e-coaching and gamification in the change process. In short, an e-coach, may enable the system to offer personalized guidance and rationalization of the behavior change process by means of a conversational experience. Gamification, on the other hand, may offer a method to introduce an immersive experience of a (virtual) future world and improved control of a particular behavior as a result of rewards and punishments. Let us now consider the two roles in more detail and in the perspective of the behavioral model and the empirical results.

### The Role of the E-Coach

Daily life is characterized by an immense degree of uncertainty, and consumption activities like SSB consumption are highly contextual in terms of location, time and company (this is underlined in Study 2). Therefore, and in line with the wishes and needs from our target group in Study 1 who sympathize with the idea of including a virtual character, a supporting agent should be highly personalized, and cautious in offering advice and solutions; what counts as a solution for one person, not necessarily counts as a solution for another. In that respect, the role of a supporting coach seems to be an excellent candidate for a digital agent that supports an automated behavior change program that fits the needs and solutions for each user individually.

In real life, coaches effortlessly align at various levels with their clients and apply a range of conversational activities to support a behavior change process and to guide their clients through the various stages of change: coaches encourage, negotiate, challenge, and explain. They also show progress, confront their clients with discrepancies in real and committed behaviors, and tailor the client's activities to the multifarious characteristics such as physical condition, age, habits, preferences, intermediary results, and experiences. In that respect, the coaching process can be considered as a continuous cycle of conversational contributions like questioning, advising, agreement, observation, feedback, and adaptation. This is also what current e-coaching systems can do.

To create a conversational experience, the e-coach may use natural language dialogue, which substantially increases the expressivity of the persuasive system. Natural language dialogue not only enables the system to explain the “why,” “when,” “what,” and “how” of activities such as self-monitoring and preparing healthy alternatives, it also functions as a persuasive instrument for personalized goal setting, constructing shared commitments, evaluation of results, and explicit motivational support as in praise and encouragement (55). The system's interface may exclude or include physical embodiment, for instance, a chat/message-style interface or a virtual character that uses both verbal and nonverbal signals. Results from Study 1 indicate that users appreciate to control at least some of the settings of the character's features in terms of language style (e.g., directivity and formality) and its appearance (e.g., gender and outfit).

Although simplistic and rigid compared to the complexity of human conversation, computer technology enables a designer of smartphone applications to offer a plethora of conversational structures that aim at behavior change. Think of protocolized sequences from motivational interviewing, acceptance and commitment theory, and structures for negotiating the properties of alternative behavior to induce some sense of autonomy – all of these strategies are key facilitators in intrinsic motivation (20, 55, 56). These conversational sequences are also the stepping-stone to further personalization of the program. To align e-coach and user, for instance, the system may simulate natural introductory sequences where both e-coach and user get acquainted to each other and build a shared model about, for instance, SSB consumption, physical conditions such as BMI, and the reasons for the detrimental behavior or the behavior change. Also, conversational feedback loops can be included to evaluate and adapt the offered recipes (see Study 1), and where shared decision making facilitates the personalization of the exercises. And last but not least, conversation introduces a wide range of social elements that create a feeling of engagement and the presence of a social partner. In particular speech acts such as welcome, praise and promise considerably contribute to the establishment and maintenance of a relationship that contributes to a feeling of trust and commitment by the user (57).

E-coach technology offers the possibility to create a persuasive system that mimics the conversational activities of an active partner who not only creates awareness about the target domain and the process of change, but who also empowers the system to offer a coherent and tailored program for change and to build a social relationship with its user.

### The Role of Gamification

To understand the importance of gamification, we should first realize that the domain of SSB consumption refers to prevention, which is fundamentally different from curing a disease or relieving the individual from a particular problem. Prevention targets at anticipatory activities (e.g., cumulative SSB consumption) before the user actually experiences a problem, such as chronic diseases developed later in life (obesity, cardiovascular diseases). Respondents in pilot study 1 and 2 indicated that the anticipatory activity delivers short-term benefits (taste, energy, quenching thirst) and outweighs uncertain long-term risk (being sick). Consequently, the individual lacks the experiential/intrinsic trigger to initiate the behavior change program, which may have important negative consequences for the motivation to adhere to supporting activities. In terms of Prochaska's transtheoretical model (41), the individual is often still in precontemplation stage where awareness should be raised about the current detrimental behavior (see also pilot study 2). In other words, why should someone take action if there is no problem, unless the action by itself is rewarding? Participants in Study 1 indicated that social media approaches might be used first to persuade individuals to start the program [c.f., Folkvord et al. (58)], but also gamification has an important role to play. Since gamification makes fun, it could be applied to lift the user to the next stage to create awareness about the need for change by making supporting activities in this process more enjoyable and challenging.

Actually, gamification elements can be implemented in all stages of the behavior change process. For instance, the monitoring process of SSB consumption aligns the system frequently and in all stages to the state of the user in terms of past and current behavior. Also, in pilot study 1 the need for education about the amount of sugar in various drinks and (the preparation of) healthy alternatives was mentioned. In that respect, supporting activities such as monitoring and education are a sine qua non for an effective intervention process, but these activities put an extra burden on the user which may dramatically decrease the adherence rate. In these cases, strategies like awards, curiosity, immersion and competition may have considerable positive impact on the user's motivation to perform various types of supporting activities. Although no strong quantitative conclusions can be drawn from the experimental pilot study, the outcome of pilot study 3 suggests that the inclusion of gamification elements has the potential to increase the chance of performing necessary supporting activities in the domain of SSB consumption, in this case self-monitoring of beverage intake. This is in line with the qualitative outcomes from the group sessions in Study 1 where the challenges-and-award version was appreciated the most. Also, education not only requires a knowledge base with information about various types of beverages, their sugar content and possibly artificial sweeteners, but also an enjoyable way of presenting the information. Hence, education may be empowered by various gaming elements such as quizzes, interactive video and simulated experimentation [see also (59)].

While the performance of supporting activities should be rewarded only by positive reinforcements, gamification may also be applied as a simulated negative reinforcer to link the user's detrimental behavior with potential future negative outcomes. This enables a designer to shift long-term outcomes of risk of excessive intake in real life to short-term consequences in a virtual environment. Since health issues may be an important motivator to quit SSB consumption (see Study 2), examples of negative reinforcers in the SSB domain can be the visualization of liver damage, type 2 diabetes consequences (e.g., medication, insulin injection, fatigue, infections, amputation), obesity or dental decay (60). This may have important consequences for the user's attitude toward SSB consumption and offers a method for persuasive strategies such as conscious raising in pre-contemplation stage and counter-conditioning in subsequent stages (39).

In addition to these negative experiences and to compensate the reduction of the positive reinforcement of the alternative behavior (replacing SSB with non-sugary healthier drinks), the system may reward intermediary achievements at the target level by the use of various types of reward mechanisms, varying from scores and unlocking mechanisms (e.g., next level, new recipes for healthy drinks) to a healthier appearance of a virtual character [c.f. Wang and Sun (61)]. It should be stressed, though, that all this requires an adequate and reliable system for monitoring the target behavior, which is, as stated before, often outside the perception of the persuasive system.

## Discussion and Conclusions

In this paper we have discussed a rationale, opportunities, basic requirements for the application of automated e-coaching and gamification as high-touch design patterns for behavior change in the domain of SSB consumption. Both design patterns could enable a persuasive system to support the user in a transformational journey from a current state to a desired state. An important task for the e-coach is to enrich the interaction with cooperative conversational experiences, in particular with respect to the achievement and maintenance of alignment between user and system, advice and feedback about the various activities, and motivational encouragement. Gamification could enable the system to expose the user at any phase in the change process to the long-term effects of the detrimental and the alternative behavior, and it improves the user's motivation to perform supporting activities by enjoyable experiences. Roughly speaking, and in terms of the dual-process theory, the e-coach triggers activities, beliefs and attitudes at the rational level of system 2, while the immersive experience of gamification has the potential to empower the process of behavior change from the conscious control of system 2 toward the habitual system 1.

To support our rationale, we applied a blended research approach targeted at adolescents using sugar sweetened beverages (SSB). Starting from the cue-behavior-outcome schema, we distinguished two types of behavior in the process of change: first, the old behavior–here the intake of SSB–that has to be replaced by alternative behavior, and second, supporting activities that have to be internalized and subsequently applied as long as the old behavior has not been replaced. Examples of supporting activities are self-monitoring of SSB consumption, e-coach conversations and education about the SSB domain. Given the sample size of our empirical studies, we should however be modest in our conclusions and these outcomes should be interpreted with caution. In a follow-up study, a larger sample should be included to test statistical significance and to study the long-term utility of gamification elements to self-monitor beverage intake. On the other hand, statistics play only a minor role in individual cases. This brings us to the next point.

The outcome of pilot study 2 indicates that SSB consumption by adolescents is related with external situational factors, such as location, time of the day and company. Therefore, both the e-coach and the gamification elements should be substantially personalized and aligned with the beliefs, the needs and the situational consumption factors of the individual. Moreover, participants mentioned that, besides taste, there are important other reasons for SSB consumption (e.g., energy boost, quenching thirst, availability) and indicated that to quit SSB consumption important other motivators besides health issues may be involved (e.g., saving money, social norm, appearance). Examples of personalization are the type and timing of rewards and the supporting activities to the location, the time and the individual's state of change [see also (62)]. The need for personalization is in line with the participants' desire to control the system's setting, in particular the timing and frequency of notifications, the gamification elements and the characteristics of the avatar.

Pilot study 1 also showed that participants sympathized with the idea of including both virtual and human characters in the application. We indeed believe that in the future the application should be part of a larger social process where virtual and human agents play a variety of competitive, normative and supportive roles, not only as a coach, but also a food expert, parent, teacher, friend or possibly even a wizard. A special role can be played, for instance, by a Tamagotchi-like agent that mirrors the user by simulating an individual that experiences the outcomes of detrimental and healthy behaviors. Human agents may play an important role in both drinking and quitting SSB consumption [c.f., (63)]. In line with Franken et al. (64), participants mentioned the role of their social network, in particular friends and social media influencers (65). It may also be recommendable to involve parents and school in the process, since infants have a natural strong preference for sweet taste and it has been observed that early introduction of added sugars in the diet may promote sweet taste preference (66–68). On the other hand, it should be mentioned that adolescents slowly turn away from their parents and friends become more important in the direct living environment, therefore, age may be an important factor for personalization. All this requires the integration of a communication system with the outside world, such as Facebook, WhatsApp or Instagram. It should be added, however, that simply comparing beverage intake with other individuals in the social prototype only had a minor, if any, effect on beverage submission. Results from the studies suggest that this strategy should be combined with an explicit reward system, such as an increase in level or status.

To conclude, current technology and in particular mobile devices offer an amalgam of persuasive strategies to initiate and support the process of behavior change. In this paper we discussed the role of two persuasive design patterns, e-coaching and gamification and their role in the behavior change process. We proposed a rationale for a gamified e-coach application to decrease the consumption of sugar sweetened beverages. Moreover, the results of the three empirical small-scale pilot studies presented in this paper provide directions and inspiration for the design and development of such application. Whereas we targeted specifically at SSB consumption, future designs or applications can incorporate similar design patterns/techniques to improve other healthy dietary habits. Future research should investigate the usability and effectiveness of the proposed gamified e-coach application and gain insight in short-term as well as long-term effectiveness of this approach in supporting healthy lifestyles.

## Data Availability Statement

The raw data supporting the conclusions of this article will be made available by the authors, without undue reservation.

## Ethics Statement

The studies involving human participants were reviewed and approved by Faculty Ethical Committee of the Department of Social Science of Utrecht University, The Netherlands. Written informed consent to participate in this study was provided by the participants' legal guardian/next of kin.

## Author Contributions

MP and RB contributed conception and design of the study and obtained funding. CL conducted the questionnaire, qualitative study, and performed the statistical analyses. CV conducted the pilot study and performed the statistical analysis. RB, MP, CL, and CV wrote sections of the manuscript. RB wrote the first and second draft of the manuscript. All authors contributed to manuscript revision, read, and approved the submitted version.

## Conflict of Interest

The authors declare that the research was conducted in the absence of any commercial or financial relationships that could be construed as a potential conflict of interest.

## Footnotes

^1^Note that the schema does not exclude conscious control in terms of intentions and reasoning.
